# Assessment of helmet usage among secondary school students in urban settings: A descriptive analytical study from Karachi, Pakistan

**DOI:** 10.1371/journal.pone.0340608

**Published:** 2026-01-09

**Authors:** Mazhar Iqbal, Kashif Shafique, Mariam Ashraf

**Affiliations:** 1 School of Public Health, Dow University of Health Sciences, Ojha Campus, Karachi, Pakistan; 2 Office of Research, Innovation & Commercialization, School of Public Health, Dow University of Health Sciences, Ojha Campus, Karachi, Pakistan; 3 Office of Research, Innovation & Commercialisation, School of Public Health, Health Services Academy, Islamabad, Pakistan; KPC Medical College and Hospital, INDIA

## Abstract

**Introduction:**

In Pakistan, the burden of road crashes continues to escalate due to inadequate traffic law enforcement and low helmet usage. This study assesses the knowledge, attitudes, and practices of adolescent motorbike riders regarding safe riding practices and helmet use.

**Methods:**

A descriptive-analytical study was conducted in public-sector secondary schools in Karachi among male students aged 15–19 years, using a structured questionnaire based on the Health Belief Model (HBM) and the Theory of Planned Behaviour (TPB). Data collection took place from October -December 2022.

**Results:**

A total of 502 students participated in the survey. The average age of the participants was 16.80 ± 1.20 years. Socio-economic data showed that most individuals belonged to the lower-middle class (63%, n = 319), with 25% (n = 125) owning personal motorcycles and 32% (n = 163) riding as passengers. The majority rode daily, with 27% (n = 137) reporting accidents without wearing helmets. Results consistent with HBM and TPB suggest a good understanding of helmet benefits but acknowledge obstacles such as helmet costs. Attitudes towards safety varied, with intentions to wear helmets immediately ranging from 24% to 29%. Lower HBM scores were more frequently observed among those from the lower-middle class (63%, n = 319) and lower socio-economic class (30%, n = 151), showing a significant association (p value = 0.002), indicating a possible disparity in knowledge and attitudes towards road safety across different socio-economic groups. Those who rode as passengers or were self-riders generally had lower HBM scores compared to others (p < 0.001).

**Conclusions:**

The findings indicated that while most students recognised that helmet use is important for safety, they demonstrated limited knowledge of proper helmet usage, traffic regulations, and other road safety measures, reflecting a gap between basic awareness and comprehensive safety knowledge.

## Introduction

Globally, road traffic accidents have been a significant concern, resulting in numerous fatalities. Around 1.19 million people died in 2021 due to road accidents. Most of these accidents happen in Low- and middle-income countries, with 90% of deaths occurring there. In the Southeast Asia Region, things are even worse, with 28% of fatalities happening there. The number of deaths of people using motorcycles and similar vehicles has gone up by 30%. Although efforts are being made to make roads safer, they are still not meeting the goal set by the UN to halve road deaths by 2030 [1]. Helmet use represents the single most effective intervention for preventing motorcycle-related head injuries, reducing the risk of death by 42% and head injury by 69% (World Health Organization, 2023). Among various risky riding behaviors, non-helmet use accounts for the highest proportion of preventable fatalities in low- and middle-income countries. The selection of helmet use as our focal behavior is based on: (1) its proven efficacy as a protective measure, (2) its modifiability through behavioral interventions, and (3) the significant gap between knowledge and practice documented in prior Pakistani studies [2].

The use of motorcycles is rapidly increasing alongside a global rise in motorisation, especially in low- and middle-income countries (LMICS) where two-wheelers serve as an affordable mode of transport for low socioeconomic groups. Motorcycles provide no protection to the rider’s body, and a crash can lead to partial disability, permanent damage to vital organs, and fatalities in severe cases. In India and Pakistan, 73% of all motor vehicles are motorcycles and motorised three-wheelers [3].

Pakistan is a developing country that suffers tremendous economic and life losses from road crashes. The fatality rate (road deaths per 10 thousand registered vehicles) in Pakistan is among the highest in the world, with more than 27 thousand people dying of crashes every year while the number of crashes is continuously increasing. The death rate in crashes in Pakistan is 14.2 per 100,000 population annually, among which more than 50% of crashes involve motorcyclists [4].

In Pakistan, although the legal minimum age for obtaining a motorcycle license is 18 years, enforcement is weak, and motorcycle use among adolescents is prevalent. Many families rely on motorcycles as the primary mode of transport, and adolescents often begin riding before reaching legal age. This reality makes it crucial to understand safety behaviors and knowledge among this age group to develop appropriate interventions [5].

Karachi, Pakistan’s largest city and economic hub with over 20 million residents, presents a critical context for this research. Pakistan ranks among countries with the highest road traffic fatality rates globally (14.2 per 100,000 population), with motorcyclists comprising over 50% of these deaths [6]. Karachi has experienced a 150% increase in motorcycle registrations since 2010, yet helmet compliance remains below 15% [7]. Unlike other major Pakistani cities, Karachi’s unique combination of dense traffic, weak enforcement infrastructure, and socioeconomic diversity makes it an ideal site for examining barriers to helmet adoption across different population segments [7].

Secondary school students (ages 15–19) represent a critical intervention window for several reasons: (1) This age group experiences the highest rate of motorcycle-related injuries in Pakistan, with 35% of motorcycle crash victims being 15–19 years old; (2) Behavioral patterns and safety attitudes formed during adolescence tend to persist into adulthood, making this a crucial period for intervention; (3) School-based settings provide systematic access to this population for educational programs; (4) In Pakistan’s context, many adolescents begin riding before the legal age of 18 due to economic necessity and weak enforcement, making early intervention essential; (5) Prior research demonstrates that interventions targeting younger riders show greater behavioral plasticity compared to adult populations. Unlike college students or working youth, secondary school students can be reached through established educational infrastructure, allowing for scalable interventions [8].

The school environment and children’s learning abilities are often seen as promising opportunities for public health improvement efforts. It is well known that knowledge and behaviours adopted early in life tend to last into adulthood. Several health initiatives have been linked to positive changes in students’ health and well-being, such as increased physical activity and fitness [9], and efforts to prevent smoking and drug abuse, among others. However, recognising that injuries are the leading cause of death among the 10–19 age group [10], there is a lack of significant published data on programmes that address risk-taking behaviours or promote safety measures, and their effectiveness.

This study integrates the Health Belief Model (HBM) and Theory of Planned Behavior (TPB) to provide a comprehensive understanding of helmet use behaviors. While both frameworks address health behaviors, they offer complementary perspectives that together provide richer insights than either model alone. The HBM focuses on individual threat perception and cost-benefit analysis, examining how perceived susceptibility to injury, perceived severity of consequences, perceived benefits of helmet use, and perceived barriers influence behaviour [11]. However, HBM does not adequately account for social influences or volitional control—critical factors in adolescent decision-making. TPB addresses these limitations by incorporating subjective norms (peer and family influences), attitudes toward the behavior, and perceived behavioral control (self-efficacy and practical constraints). In the context of adolescent helmet use, where peer pressure and parental modeling significantly influence behavior, TPB’s social components are essential. The integration allows us to examine: (1) individual risk perceptions (HBM) alongside social influences (TPB); (2) practical barriers like cost and comfort (HBM barriers) in conjunction with perceived control over helmet access (TPB); and (3) both cognitive threat appraisal (HBM) and attitudinal factors (TPB). This combination has been successfully used in previous adolescent safety research and aligns with the socio-ecological model of health behavior, which recognizes that adolescent decisions result from both individual cognitions and social contexts.

## Materials and methods

### Study design, participants, and sampling strategy

This study is a descriptive analytical study. This study was conducted in public-sector secondary schools in both higher- and lower-socioeconomic communities. Data was collected from male students attending public schools. Recruitment for the study took place between October 2022 and December 2022.

In Pakistan’s public education system, significant age variation exists within secondary grades due to several factors: (1) late school entry, particularly in lower socioeconomic families where children may start school at 7–8 years instead of 5–6 years; (2) grade repetition due to academic failure or prolonged absences; (3) interrupted schooling due to family financial crises or migration. According to Pakistan Education Statistics 2022 [12], approximately 23% of students in grades 9–10 in public schools are 17 years or older. Our inclusion of 15–19-year-olds reflects this educational reality and captures the age group at highest risk for motorcycle injuries. We documented each participant’s current grade level and confirmed enrollment through school records. Age distribution was: 15 years (n = 89, 17.7%), 16 years (n = 142, 28.3%), 17 years (n = 156, 31.1%), 18 years (n = 87, 17.3%), 19 years (n = 28, 5.6%).

The sample size was calculated using Openepi.com with a two means comparison formula. The mean ± SD of knowledge about helmet use was 7.16 ± 1.55 in the intervention group and 6.84 ± 1.68 in the control group, with a 95% confidence interval and 80% study power [13]. The initial calculated sample size was 416, but it was increased to 500 to account for a potential 20% non-response rate or incomplete questionnaires. However, we achieved a 100% response rate with complete data from all 502 participants, as data collection was conducted during school hours with proper consent procedures. The number of clusters (schools) and cluster size (students) were determined with STATA software, considering an intra-cluster correlation coefficient of 0.05 to account for variance within clusters, with 80% power and a 95% confidence level. This resulted in 5 clusters per arm (10 schools total), each with 50 students. Stratified cluster sampling was conducted (using computer-generated random numbers) from seven districts and public-sector secondary schools involved in the study. School identifiers or other unique codes assigned to students in grades 8–10 and aged 15–19 years using a motorcycle for transport as a rider, pillion rider, or passenger served as a sampling frame. A computer-generated random number was then used to select students for interviews after receiving formal parental consent.

### Ethical considerations

This research was approved by the institutional review board IRB-2510/DUHS/Approval/2022/ 856. The selection of schools was made with prior notification and approval from the Sindh Education Department. A formal request for a detailed list of schools with enrolment figures was sent to the education department after obtaining institutional ethical clearance. All 15–19 year-old male students attending secondary public schools who consented and used a motorbike for transportation, either as a passenger or pillion rider, were included in the study. Written consent (in Urdu) was obtained from the participants/parents in accordance with the Declaration of Helsinki. For students under the age of 18, written parental/guardian consent was obtained before data collection, in accordance with the ethical guidelines outlined in the Declaration of Helsinki. Participation was entirely voluntary, and anonymity and confidentiality of the respondents were ensured throughout the study.

### Survey instrument

The questionnaire was systematically developed through a three-phase process:

**Phase 1** – Item Generation: We adapted validated HBM scales from Champion & Skinner (2008) [14] and TPB measures following Ajzen’s (2006) [15] construction guidelines. Specifically, HBM items assessing perceived susceptibility, severity, benefits, and barriers were adapted from Champion’s breast health studies and modified for motorcycle safety context through expert review by three road safety researchers and two behavioral psychologists. TPB items measuring attitudes, subjective norms, perceived behavioral control, and intentions were constructed following Ajzen’s recommended question format, with adaptations for adolescent comprehension level and cultural context. Knowledge items were developed based on Pakistan’s National Highway and Motorway Police road safety curriculum [16].
**Phase 2** – Content Validation: The initial 65-item instrument underwent content validation by a panel of 5 experts (2 road safety specialists, 2 health behavior researchers, 1 adolescent psychologist). Content validity index was 0.89. The questionnaire was then translated into Urdu using forward-backward translation methodology, with discrepancies resolved through expert consensus.
**Phase 3** – Pilot Testing and Psychometric Evaluation: The questionnaire was pilot-tested with 50 students from non-study schools. Factor analysis (KMO = 0.83, Bartlett’s test p < 0.001) confirmed the theoretical structure. Principal component analysis with varimax rotation revealed 11 distinct factors corresponding to theoretical constructs, explaining 64% of total variance. Factor loadings for all items exceeded 0.40. Internal consistency was assessed using Cronbach’s alpha: Knowledge scale (α = 0.76), HBM subscales (perceived susceptibility α = 0.81, perceived severity α = 0.78, perceived benefits α = 0.84, perceived barriers α = 0.79, cues to action α = 0.77, self-efficacy α = 0.82), TPB subscales (attitudes α = 0.85, subjective norms α = 0.80, perceived behavioral control α = 0.83, intentions α = 0.88). Test-retest reliability assessed with 30 students after 2 weeks showed intraclass correlation coefficients ranging from 0.76 to 0.89.

### Operational definitions

Accident history was assessed through the question: ‘In the past 12 months, have you been involved in any motorcycle accident either as a rider or passenger?’ Response options were: (1) Never had an accident while riding, (2) Never had an accident while sitting as a passenger, (3) Had an accident while wearing a helmet, (4) Had an accident while not wearing a helmet. Participants who had multiple accidents in different scenarios were instructed to select the most recent incident.

Accidents were defined as ‘any collision or crash involving a motorcycle that resulted in injury requiring medical attention or property damage.’ Helmet use frequency was assessed separately for riding and passenger situations: ‘How often do you wear a helmet when you ride a motorcycle?’ and ‘How often do you wear a helmet when you sit as a passenger?’ Response options used a 5-point scale: Always (100% of the time), Often (75–99%), Sometimes (26–74%), Rarely (1–25%), Never (0%). For analytical purposes, these were collapsed into three categories: Always, Often/Sometimes/Rarely, and Never.

Socioeconomic status was assessed using the modified Kuppuswamy scale adapted for Pakistan [17]. This composite index assigns scores based on three components: (1) family head’s education (1–7 points), (2) family head’s occupation (1–10 points), and (3) total monthly family income (1–12 points). Total scores range from 3–29, classified as: Upper class (26 –29), Upper-middle class (16–25), Lower-middle class (11–15), Lower class (5–10), Below poverty line (<5). For our analysis, we collapsed categories into three groups due to sample distribution: Middle class (scores 16–25, n = 32), Lower-middle class (scores 11–15, n = 319), Lower class (scores ≤10, n = 151). Income brackets were adjusted for 2022 Pakistani rupee values and urban Karachi cost of living. Cutoff scores for ‘high’ and ‘low’ categories were determined using the median split approach.

Knowledge scores ranged from 3–16 (median = 11, IQR: 9–13). We classified scores ≥11 as ‘adequate knowledge’ and <11 as ‘inadequate knowledge,’ aligning with the educational standard of ≥70% correct responses indicating passing performance. HBM total scores ranged from 12–33 (median = 23, IQR: 19–26). Scores ≥23 were classified as ‘high HBM’ and <23 as ‘low HBM.’ TPB total scores ranged from 8–22 (median = 11, IQR: 9–14). Scores ≥11 were classified as ‘high TPB’ and <11 as ‘low TPB.’

### Data management, collection and analysis

The data collection process for this study was comprehensive and well-organised. Using the pre-designed and tested questionnaire, the data collection team ensured the collection of information according to the study design and schedule. The filled questionnaires were submitted to the principal investigator on the same day after each session/intervention and were kept in a locked storage file before and after data entry in the computer. The storage file was kept secure in a safe place in the office and access was restricted.

Data analysis was conducted using IBM SPSS version 27. Descriptive statistics (frequencies, percentages, means, and standard deviations) were calculated for demographic characteristics and study variables. Pearson chi-square tests were used to examine the associations between categorical variables (socioeconomic status, transport characteristics, and helmet usage patterns) and knowledge/HBM/TPB scores. A p-value of <0.05 was considered statistically significant.

## Results

### Socio-demographic characteristics

A total of 502 students aged 15–19 years were interviewed. The mean age of the participants was 16.80 ± 1.20 years. The majority of the students belonged to the lower-middle class group, 63% (n = 319), followed by 30% (n = 151) to the lower class group, and only 6% (n = 32) belonged to the middle class (Table 1). Among the students, 25% (n = 125) reported owning a personal motorbike and riding it, representing the largest single category of motorcycle users. Regarding past experiences with accidents, 46% (n = 231) of students reported never having had an accident while riding, while 27% (n = 137) reported having an accident while not wearing a helmet (Table 1).

**Table 1:** Demographic Characteristics and Use of Motorbikes.

Characteristics	N = 502 (%)
**Demographic**	
**Age (years), mean ± SD**	16.80 ± 1.20
**Socio-economic status**	
Middle Class	32 (6.4)
Lower – middle Class	319 (63.5)
Lower Class	151 (30.1)
**Type of Motorbike User**	
Own a personal motorbike/ Ride bike myself	125 (24.9)
Own a personal motorbike/ Ride bike myself/Driving license holder	89 (17.7)
Driving license holder	83 (16.5)
Don’t ride myself but sit as a passenger on a motorbike	163 (32.5)
Don’t own or ride a motorbike/don’t have a license	42 (8.4)
**Riding Frequency**	
Once a Month or less	44 (8.8)
Two to three times a month	33 (6.6)
Once or twice a week	45 (9.0)
At least thrice a week	77 (15.3)
Every day or almost every day	303 (60.3)
**History of Accident**	
Never had an accident while riding a bike	231 (46.0)
Never had an accident while sitting on a bike	102 (20.3)
Had an accident while wearing a helmet	32 (6.4)
Had an accident while not wearing a helmet	137 (27.3)

### Distribution of demographic and transport characteristics according to knowledge, HBM scores and TPB scores of children

Table 2 displays demographic and transport characteristics distribution based on participant knowledge, HBM scores, and TPB scores regarding road safety. The majority of individuals with lower HBM scores were from the lower-middle class (63%, n = 283) and the lower class (32%, n = 141), showing a strong association between these groups and their HBM scores (*p* value = 0.002). The study found no significant association between the participants’ knowledge and TPB scores with demographic variables. Regarding the different types of motorbike users, individuals who rode their bikes as passengers (31%, n = 139) and those who were owners or self-riders (28%, n = 123) had lower HBM scores than those in other groups. The type of motorcycle riders and HBM predictors were strongly associated (p-value <0.001). Furthermore, compared to those using 70cc motorbikes or who were unsure about the type of motorbike, 50% (n = 225) of those using 100cc motorbikes had lower HBM ratings, indicating a significant association between the range of motorcycles and HBM indicators. Similarly, a significant association was recorded between the range of motorcycles and TPB scores (*p* value = 0.016). Participants using 100cc motorbikes (n = 219, 44%) recorded the lowest TPB scores.

**Table 2:** Distribution of demographic and transport characteristics according to knowledge, HBM scores and TPB scores of children.

Demographic and Transport Characteristics	Inadequate Knowledge	Adequate Knowledge	*P-*value^š^	HBM Low score	HBM High score	*P-*value^š^	TPB score Low	TPB High score	*P-*value^š^
	n=380 (%)	n=122 (%)		n=447 (%)	n=55 (%)		n=423 (%)	n=79 (%)	
**Age**									
Below 18 years	283 (74.5)	92 (75.4)	0.836	333 (74.5)	42 (76.4)	0.764	311 (62.0)	64 (12.7)	0.160
> 18years	97 (25.5)	30 (24.6)		114 (25.5)	13 (23.6)		112 (22.3)	15 (3.0)	
**Socio-economic status**									
Middle Class	24 (6.3)	8 (6.6)		23 (5.1)	9 (16.4)		25 (5.0)	7 (1.4)	
Lower – middle Class	234 (61.6)	85 (69.7)	0.213	283 (63.3)	36 (65.5)	0.002	268 (53.4)	51 (10.2)	0.521
Lower Class	122 (32.1)	29 (23.8)		141 (31.5)	10 (18.2)		130 (25.9)	21 (4.2)	
**Way of transport to school**	
Motorbike (Self riding)	210 (55.3)	64 (52.5)		249 (55.7)	25 (45.5)		239 (47.6)	35 (7.0)	
Motorbike rider as passenger	78 (20.5)	29(23.8)	0.742	92 (20.6)	15 (27.3)	0.328	90 (17.9)	17 (3.4)	0.056
Others^7^	92 (24.2)	29 (23.8)		106 (23.7)	15 (27.3)		94 (18.7)	27 (5.4)	
**Type of motorbike user**									
Motorbike owner/ Biker rider	94 (24.7)	31 (25.4)		123 (27.5)	2 (3.6)		106 (21.1)	19 (3.8)	
Motor bike owner/ Bike rider/license holder	71 (18.7)	18 (14.8)		77 (17.2)	12 (21.8)		77 (15.3)	12 (2.4)	
Driving license holder	59 (15.5)	24 (19.7)	0.521	67 (15.0)	16 (29.1)	<0.001	67 (13.3)	16 (3.2)	0.878
Rider as a passenger	127(33.4)	36 (29.5)		139 (31.1)	24 (43.6)		138 (27.5)	25 (5.0)	
Don’t own or ride bike/don’t have license	29 (7.6)	13 (10.7)		41 (9.2)	1 (1.8)		35 (7.0)	7 (1.4)	
**Riding Frequency**									
Once a Month or less	38 (10.0)	6 (4.9)		40 (8.9)	4 (7.3)		39 (7.8)	5 (1.0)	
Two to three times a month	22 (5.8)	11 (9.0)		27 (6.0)	6 (10.9)		26 (5.2)	7 (1.4)	
Once or twice a week	35 (9.2)	10 (8.2)	0.20	39 (8.7)	6 (10.9)	0.643	33 (6.6)	12 (2.4)	0.207
At least thrice a week	62 (16.3)	15 (12.3)		70 (15.7)	7 (12.7)		66 (13.1)	11 (2.2)	
Every day or almost every day	223 (58.7)	80 (65.6)		271 (60.6)	32 (58.2)		259 (51.6)	44 (8.8)	
**Type of motorbike**									
70 cc	146 (38.4)	46 (37.7)		181 (40.5)	11 (20.0)		165 (32.9)	27 (5.4)	
100 cc and above	198 (52.1)	57 (46.7)	0.159	225 (50.3)	30 (54.5)	<0.001	219 (43.6)	36 (7.2)	0.016
Don’t know the type of motorbike	36 (9.5)	19 (15.6)		41 (9.2)	14 (25.5)		39 (7.8)	16 (3.2)	
**History of Accident and History of Fine**									
Never had an accident while riding a bike	179 (47.1)	52 (42.6)		211 (47.2)	20 (36.4)		196 (39.0)	35(7.0)	
Never had an accident while sitting on a bike	70 (18.4)	32 (26.2)	0.323	84 (18.8)	18 (32.7)	0.097	81 (16.1)	21 (4.2)	0.098
Had an accident while I was wearing helmet	25 (6.6)	7 (5.7)		28 (6.3)	4 (7.3)		24 (4.8)	8 (1.6)	
Had an accident while I was not wearing helmet	106 (27.9)	31 (25.4)		124 (27.7)	13 (23.6)		122 (24.3)	15 (3.0)	

šPearson chi-square test, ^7^Others = Bicycle, Vehicle, Public Transport, School Van, Pedestrian, HBM: Health Belief Model, TPB: Theory of Planned Behavior.

Table 3 presents data on various characteristics and behaviours related to helmet usage among individuals, divided into groups with inadequate knowledge and adequate knowledge based on the Health Belief Model (HBM) and Theory of Planned Behaviour (TPB) scores. The participants who rarely wore helmets (48%, n=214), or never wore helmets (41%, n=182), while riding motorbikes, were more with lower HBM scores compared to participants with high HBM scores. However, a strong association was found between helmet use while riding a motorbike and HBM scores (p-value = 0.013). A significant association was found between passengers who never wore helmets and their HBM scores (p-value = 0.043). The participants who never wore helmets as passengers were more (47%, n=210), with low HBM scores compared to participants with high HBM scores. Regarding participants’ siblings’ helmet use for road safety, participants reported that their brothers rarely wore helmets (51%, n=192), and this was strongly associated with inadequate knowledge about road safety measures (p-value = 0.019). Participants’ friends’ helmet use for road safety was significantly associated with low HBM scores. Specifically, (51%, n=229) of participants reported that their friends rarely wore helmets, and (n=179, 40%) reported that their friends never wore helmets. (41%, n=208) of the participants with lower TPB scores reported that people rarely wore helmets with whom they sat for a ride on motorbikes, and this association was significant (p-value = 0.032). Interestingly, participants who scored inadequate knowledge and low HBM scores about road safety indicated a willingness to wear helmets while riding motorbikes next time, (37%) always or (42%) sometimes. This association was statistically significant (p-value<0.001)

**Table 3:** Distribution of type of helmet user according to Knowledge, HBM scores and TPB scores of children.

Characteristics	Inadequate Knowledge n = 380 (%)	Adequate Knowledge n = 122 (%)	*P-*value^š^	HBM Low score n = 447 (%)	HBM High score n = 55 (%)	*P-*value^š^	TPB Low score n = 423 (%)	TPB High score n = 79 (%)	*P-*value^š^
**Wear helmet when ride a bike**									
Always	45 (11.8)	20 (16.4)	0.092	51 (11.4)	14 (25.5)	**0.013**	51 (10.2)	14 (2.8)	0.160
Often/sometimes/Rarely	188 (49.5)	47 (38.5)		214 (47.9)	21 (38.2)		195 (38.8)	40 (8.0)	
Never	147 (38.7)	55 (45.1)		182 (40.7)	20 (36.4)		177 (35.3)	25 (5.0)	
**Wear helmet when sit as a passenger**									
Always	25 (6.6)	15 (12.3)	0.127	31 (6.9)	9 (16.4)	**0.043**	30 (6.0)	10 (2.0)	0.243
Often/sometimes/Rarely	178 (46.8)	53 (43.4)		206 (46.1)	25 (45.5)		197 (39.2)	34 (6.8)	
Never	177 (46.6)	54 (44.3)		210 (47.0)	21 (38.2)		196 (39.0)	35 (7.0)	
**Brother usually wear helmet**									
Always	56 (14.7)	28 (23.0)	**0.019**	71 (15.9)	13 (23.6)	0.348	68 (13.5)	16 (3.2)	0.455
Often/sometimes/Rarely	192 (50.5)	66 (54.1)		32 (51.9)	26 (47.3)		216 (43.0)	42 (8.4)	
Never	132 (34.7)	28 (23.0)		144 (32.2)	16 (29.1)		139 (27.7)	21 (4.2)	
**Friends usually wear helmets**									
Always	37 (9.7)	14 (11.5)	0.783	39 (8.7)	12 (21.8)	**0.009**	42 (8.4)	9 (1.8)	
Often/sometimes/Rarely	192 (50.5)	63 (51.6)		229 (51.2)	26 (47.3)		219 (43.6)	36 (7.2)	0.598
Never	151 (39.7)	45 (36.9)		179 (40.0)	17 (30.9)		162 (32.3)	34 (6.8)	
**Person wear helmet with whom I sit**									
Always	63 (16.6)	27 (22.1)	0.337	75 (16.8)	15 (27.3)	0.104	68 (13.5)	42 (4.4)	**0.032**
Often/sometimes/Rarely	181 (47.6)	57 (46.7)		212 (47.4)	26 (47.3)		208 (41.4)	30 (6.0)	
Never	136 (35.8)	38 (31.1)		160 (35.8)	14 (25.5)		147 (29.3)	27 (5.4)	
**Intend to wear a helmet the next time**									
Always	137 (36.1)	73 (59.8)	**<0.001**	167 (37.4)	43 (78.2)	**<0.001**	168 (33.5)	42 (8.4)	0.084
Often/sometimes/Rarely	161 (42.4)	35 (28.7)		186 (41.6)	10 (18.2)		171 (34.1)	25 (5.0)	
Never	82 (21.6)	14 (11.5)		94 (21.0)	2 (3.6)		84 (16.7)	12 (2.4)	

^**š**^ Pearson chi-square test, HBM: Health Belief Model, TPB: Theory of Planned Behaviour.

## Discussion

The study findings revealed a significant lack of awareness among the surveyed students regarding road safety rules and regulations, particularly regarding motorcycle helmet use. The strong association between lower socioeconomic status and reduced HBM scores reflects multiple interconnected mechanisms. First, economic constraints create tangible barriers. Helmet costs (PKR 500–2000, equivalent to 3–14 days of minimum wage income) represent substantial expenses for lower-income families, who must prioritise basic necessities. This aligns with previous research in Bangladesh and India, which shows cost as the primary barrier to helmet adoption among lower-SES populations [18,19]. Second, lower SES correlates with reduced access to safety education, as schools in lower-income areas typically lack road safety programs. Third, enforcement disparities exist, with traffic police focusing enforcement in wealthier neighborhoods, creating differential normative pressures. These findings underscore that helmet promotion cannot rely solely on education but must address structural economic barriers through subsidy programs or free helmet distribution [20,21].

Our finding that ‘siblings’ and ‘friends’ helmet behaviours significantly predict participants’ own practices aligns with Social Learning Theory and TPB’s subjective norms construct. Adolescents in collectivist cultures like Pakistan particularly rely on peer referents for behavioral guidance. The strong peer influence suggests that interventions targeting individual riders will have limited effectiveness without addressing social networks. Successful approaches might include peer education programs, visible role modeling by influential figures, and whole-school campaigns that shift normative perceptions.

The complementary insights from HBM and TPB validate our integrated approach. HBM effectively identified cognitive barriers (cost, discomfort) and threat perceptions, while TPB revealed the powerful role of social norms and perceived control. Interestingly, participants demonstrated awareness of helmet benefits (HBM perceived benefits) yet reported intentions not to wear helmets, with TPB variables (particularly subjective norms and perceived control) explaining this attitude-behavior gap. This suggests that threat awareness alone is insufficient; interventions must enhance social support and self-efficacy while reducing practical barriers.

Our findings parallel studies from Vietnam and Thailand showing socioeconomic gradients in helmet use [22] but contrast with Western contexts where enforcement is the primary driver. This suggests that interventions must be context-specific, addressing local economic realities and social structures rather than simply importing Western enforcement models.

Additionally, this study identified a strong association between the type of motorbike users and their HBM scores those who rode as passengers or were self-riders tended to have lower HBM scores compared to other groups. Furthermore, participants using 100cc motorbikes showed lower HBM (p < 0.001). and TPB scores (p = 0.016), suggesting a potential link between the type of motorcycle used and safety perceptions. This implies that riders of lower-powered motorcycles might perceive risks and safety differently compared to those using more powerful bikes. Different studies discussed the possible interactions between the type of driven motorcycle (different classes and/or engine capacities) and helmet usage [23]. In Skalkidou and colleagues’ study [24], there was a significant relationship between the engine size category and helmet usage (14.3% for engines less than 50 cc, 12.6% for 51–200 cc, 31.8% for 201–400 cc, and 32.5% for greater than 400 cc engines), in which an increase in the category of the engine had an OR of 1.52 for helmet usage (95% CI = 1.30–1.75, P = 0.0001).

Furthermore, students with inadequate knowledge and low HBM scores expressed a willingness to wear helmets in the future, suggesting the potential impact of educational campaigns on attitudes and safer practices. A similar study conducted in Iran concluded that, using the TPB, drivers with more positive attitudes were more likely to have intentions and perceptions of safe driving. The study also recommended that programs to improve attitudes should be designed to reduce road traffic injuries [25].

However, barriers such as perceived costs and comfort issues were reported, emphasising the importance of addressing these practical concerns to facilitate the widespread adoption of helmet-wearing behaviours. In Iribhogbe and Odai’s study, they found that many motorcyclists complain of the cost of helmets, while many had helmets but refused to wear them due to “inconveniencies” [26]. Despite overwhelming evidence that helmet use prevented head trauma [27]. Drawing comparisons with international studies focusing on youth helmet usage and road safety can provide valuable insights into best practices and effective strategies that have been implemented in other global contexts. Research from countries such as Australia, the United States, and European nations has highlighted successful interventions and policies that have increased helmet use among adolescents and young adults [28].

These findings endorse multi-level interventions: (1) Economic strategies like helmet subsidies or free distribution programmes targeting low-SES populations; (2) Educational initiatives: school-based programmes that fill knowledge gaps, build self-efficacy, and challenge peer norms; (3) Environmental strategies: better enforcement with education-first approaches rather than punitive measures that may unfairly burden low-SES riders; (4) Social marketing: campaigns featuring peer role models and tackling comfort and style concerns identified as barriers.

This study’s strengths include its theory-driven approach, large sample size, validated instruments, and inclusion of diverse socioeconomic groups. However, several limitations warrant consideration. First, the cross-sectional design precludes causal inference—we cannot determine whether low HBM scores lead to non-helmet use or whether non-use shapes cognitive appraisals. Longitudinal studies tracking behavior change are needed. Second, self-reported behaviors may be subject to social desirability bias, though we attempted to minimize this through trained interviewers and assured anonymity. Observational studies would strengthen findings. Third, interviewer-administered data collection, while necessary for ensuring comprehension across varying literacy levels, may have introduced interviewer effects despite training. Fourth, our sample from public schools in Karachi may not represent private school students or rural populations, limiting generalizability. Fifth, we did not assess helmet quality or proper usage, which affect actual protection. Sixth, unmeasured factors like traffic density exposure, previous near-misses, or family motorcycle crash history may confound observed associations. The inclusion of 42 non-riders (8.4%), while providing comparative insights, may have diluted effect sizes. Finally, recall bias may affect accident history reporting, though restricting recall to 12 months should minimize this issue.

Future studies should: (1) Conduct longitudinal research tracking behavior change over time; (2) Implement and evaluate theory-based interventions combining education, economic support, and social marketing; (3) Examine differential intervention effectiveness across socioeconomic groups; (4) Assess quality and proper use of helmets, not just ownership; (5) Explore family dynamics and parental modeling effects; (6) Investigate gender differences through studies including female riders and passengers.

## Conclusion

This study of 502 adolescent students in Karachi revealed critical gaps between awareness and practice of helmet usage. While most students recognised the importance of helmets, only a minority consistently wore them. Lower socioeconomic status and specific motorcycle user types (passengers and self-riders) were associated with lower HBM scores, indicating disparities in safety behaviours. Social influences (siblings and peers) significantly affected helmet use decisions. Targeted interventions addressing socioeconomic barriers, peer norms, and practical concerns (cost, comfort) are needed. School-based education programmes combined with improved enforcement and community engagement can effectively promote helmet usage among adolescent motorcyclists in urban Pakistan.

## Supporting information

S1 Checklist Strobe Checklist for the cross-sectional study. (DOCX)

S2 Checklist Plos One Human subject research checklist. (DOCX)

S1 File Questionnaire. (PDF)

S2 File Consent form. (DOCX)

## Abbreviations

LMICs: Low and Middle- Income Countries
TPB: Theory of Planned Behaviour
HBM: Health Belief Model
PI: Principal Investigator
SPSS: Statistical Package for the Social Sciences
SD: Standard Deviation
OR: Odds Ratio
CI: Confidence Interval
