# Supplementary material for: Assessment of helmet usage among secondary school students in urban settings: A descriptive analytical study from Karachi, Pakistan
Source: PLoS One. 2026 Jan 9;21(1):e0340608. doi: 10.1371/journal.pone.0340608 (PMC12788624; doi:10.1371/journal.pone.0340608)
Supplement: S1 Checklist — (DOCX) [file pone.0340608.s001.docx]

STROBE Checklist – Cross-Sectional Study

| **Section** | **Item No.** | **STROBE Recommendation** | **Addressed in the Paper** |
| --- | --- | --- | --- |
| Title and Abstract | 1a | Indicate study’s design in title or abstract | ✔ Mentioned as "cross-sectional survey" in both |
|  | 1b | Informative and balanced summary of what was done/found | ✔ Abstract summarizes methods, key findings, and conclusions |
| Introduction | 2 | Explain scientific background/rationale | ✔ Discusses global and local road safety challenges and theoretical basis |
|  | 3 | State specific objectives/prespecified hypotheses | ✔ Clear objectives stated to assess knowledge, attitudes, and practices |
| Methods | 4 | Present key elements of study design early | ✔ Identified as quantitative cross-sectional |
|  | 5 | Describe setting, locations, relevant dates | ✔ Conducted in Karachi, with description of school selection and permissions |
|  | 6a | Eligibility criteria, source and method of selection | ✔ Male students aged 15–19, random selection using school lists |
|  | 7 | Define all outcomes, exposures, confounders | ✔ Knowledge, HBM/TPB scores, socio-demographic data all defined |
|  | 8* | Sources of data and measurement methods | ✔ Structured validated questionnaire described with translation/back-translation |
|  | 9 | Describe efforts to address bias | ✔ Random selection, pilot testing, face validity, Cronbach’s Alpha discussed |
|  | 10 | Explain how study size was determined | ✔ Sample size calculated using OpenEpi and STATA, with ICC and attrition adjustment |
|  | 11 | Handling of quantitative variables | ✔ Cutoffs for HBM/TPB and knowledge scores explained; scores treated categorically |
|  | 12a | Statistical methods incl. confounding control | ✔ Chi-square test used, p-values reported for associations |
|  | 12b | Subgroup/interactions methods | ✔ Subgroups by SES, bike use, accident history examined |
|  | 12c | Missing data addressed | ✔ Implied complete data with 100% response rate, no discussion on missing data |
|  | 12d | Analytical methods accounting for sampling | ✔ Cluster sampling with ICC considered in design stage |
|  | 12e | Sensitivity analyses | ❌ Not reported |
| Results | 13a | Numbers at each stage of study | ✔ 502 students included; 100% response rate noted |
|  | 13b | Reasons for non-participation | ✔ No refusals reported |
|  | 13c | Flow diagram | ❌ Not included |
|  | 14a | Participant characteristics | ✔ Table 1: age, SES, transport type, etc. |
|  | 14b | Missing data per variable | ❌ Not reported |
|  | 15* | Outcome events or summary measures | ✔ Knowledge, HBM, and TPB scores reported in detail |
|  | 16a | Unadjusted and adjusted estimates with CIs | ❌ Only p-values for chi-square tests reported, no CIs or adjusted models |
|  | 16b | Category boundaries for variables | ✔ Cutoffs and categorizations (e.g., HBM ≥23 = high) provided |
|  | 16c | Translation of risks into absolute terms | ❌ Not applicable/reported |
|  | 17 | Other analyses (subgroups, sensitivity) | ✔ Subgroups by SES, riding behavior, motorbike type analyzed |
| Discussion | 18 | Key results summarized with objectives | ✔ Yes |
|  | 19 | Limitations discussed | ✔ Discussed self-reporting bias, generalizability, and lack of observation |
|  | 20 | Interpretation of results | ✔ Results interpreted in light of objectives and literature |
|  | 21 | Generalisability discussed | ✔ Addressed; mentions findings are not nationally representative |
| Other Information | 22 | Source of funding and funders’ role | ✔ Acknowledged support from Education Dept., no specific funding agency |
