# Supplementary material for: Assessment of helmet usage among secondary school students in urban settings: A descriptive analytical study from Karachi, Pakistan
Source: PLoS One. 2026 Jan 9;21(1):e0340608. doi: 10.1371/journal.pone.0340608 (PMC12788624; doi:10.1371/journal.pone.0340608)
Supplement: S1 File — (PDF) [file pone.0340608.s003.pdf]

Assessment of Helmet Usage Among Secondary School Students In Urban Settings Of Karachi, Pakistan

CONFIDENTIAL INFORMATION: TO BE USED FOR RESEARCH AND POLICY PURPOSES ONLY

SECTION I DEMOGRAPHIC PROFILE

(To be filled in by all respondents)

Q1a. Record the gender.(Single choice)

Male	1
Female	2
Other	3

Q1b.May I have your date of birth? ______(year)_______(month) ________(day)

Interviewer should calculate age (in completed years) from date of birth and write. _______

Q1c (single choice) What is the highest level of your head of family (tick any one : father's OR mother's) education received? Ask for completed education.

Masters or above (PhD, MS, MD)	7
University Graduate	6
Intermediate (11-12 grade)Secondary school	5
High school (9-10 grade)	4
Middle School (6-8 grade)	3
Primary school	2
No Education / No formal schooling/ Illiterate	1

Q1d (single choice) What is occupation of your father?

Profession (Doctor/engineer/lawyer/Politician/Businessman	10
Semi profession/ Employee private sector (social work, journalism, librarianship, te aching and nursing)	6
Clerical/Shop owner	5
Skilled worker (law enforcement officers, financial technicians, nurses, sales repres entative, and electricians	4
Semi-Skilled worker (drivers, retail salespersons, flight attendants, taxi drivers, waiters, and security guards)	3
Daily wager/Unskilled worker (grocery clerks, maids, fast food workers, janitors, and parking lot attendants)	2
Un employed	1

Q1e Please tick about the family income of your family (father's/mother's or both) (single choice)

PKR	Family income /month
> 39377	12
19606 - 39376	7
14700 - 19605	6
9794 - 14699	4
5869 - 9793	3
1964 - 5868	2
< 1963	1

Q2a (single choice) What is your preferred way of transport to school ?

Motorbike (Self riding)	1
Motorbike as passenger with father/family member/friend	2
Pedestrian	3
Bicycle	4
Vehicle (personal/shared with/by friend	6
Public Transport	7
School Van	8
Please provide make/registration of motorbike	

Q2bType of Motorbike User

	Yes	No
I own a personal motorbike	1	2
I ride bike myself	1	2
I own a helmet	1	2
I have a driving license	1	2
I don't ride myself but sit as a passenger on motorbike	1	2

Q2c.Riding Frequency

	Code
Once a Month or less	1
Two to three times a month	2
Once or twice a week	3
At least thrice a week	4
Every day or almost every day	5

Q2d.Type of motorbike (Multiple responses accepted)

Which type of motorbike you use?,	Yes	No
70 cc	1	2
100 cc and above	1	2
Don't know	1	2
Other		

Q2e. History of Accident and History of Fine

	Code
I Never had an accident while riding a bike	1
I never had an accident while sitting on a bike	2
I had an accident while I was wearing helmet	3

I had an accident while I was not wearing helmet	4

Qf Type of helmet user

	Always	Often	Sometimes	Rarely	Never
Do you wear helmet when ride a bike	1	2	3	4	5
Do you wear helmet even when sit as a passenger	1	2	3	4	5
Do any of your brothers usually wear bike helmets when they ride?	1	2	3	4	5
Do your friends usually wear bike helmets when they ride?	1	2	3	4	5
The person I sit as a passenger wear helmet	1	2	3	4	5
Do you intend to wear a helmet the next time you ride a bike?	1	2	3	4	5
Do you currently use motorbike for school transportation?	1	2	3	4	5

KNOWLEDGE INDICATORS

Now I am going to ask you few questions about road safety

Q3a

**What does a broken white line mean?**	Code
Never cross it	1
Overtake if it's safe to do so	2
Stay in your own lane at all times	3
Walk slowly	4

Q3b

**Driver's who are talking on a cellular phone are**	Code
Alert and aware of their surroundings	1
Focused on the task of driving	2
Distracted and more likely to get in an accident	3

Q3c

**What does this sign mean?** 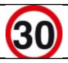	Code
Average speed that cars are traveling at	1
Minimum speed you should drive at	2
Speed that you should drive if it is raining	3
Maximum speed you should drive at	4

Q3d

A flashing red light at an intersection means you should	Code
Stop completely and check all other roads coming into the intersection before moving forward.	1
Proceed with caution	2
Wait for a green light	3
Ignore the light completely and continue moving forward	4

Q3e

The rules for motorbike riders include:	YES	NO	Don't Know
While riding on cycle or bike, both the riders must wear helmets.	1	2	95
Music is not played (headphones)	1	2	95
Mobile phone is not used during riding a bike.	1	2	95
The biker must not smoke during a ride.	1	2	95
The bikers do not ride fast and recklessly.	1	2	95
The biker follows the traffic signs for a safe ride.	1	2	95

Q3f

	YES	NO	Don't Know
I have heard about road safety	1	2	95
It is permissible for anyone to wear a headset while driving.	1	2	95
A driver may pass another vehicle by driving on the shoulder of the road.	1	2	95
Over speeding is the most important cause of fatalities in Pakistan?	1	2	95
I know that Use of helmet protect my head	1	2	95
I know that Use of helmet can prevent from injuries/disabilities	1	2	95

HBM (Predictors)

How strongly do you disagree or agree with the following statements about the impact of seeing or hearing messages on road safety and helmet wearing.**(READ OUT):**
The messages…

Please answer all questions by putting corresponding number from the answer options. Do not leave any question
Answer only one option in each scale
Q4a. Personal Vulnerability Perceived Exemption From Harm

	**Disagree Completely 1**	**Somewhat disagree 2**	**Neither agree, nor disagree 3**	**Somewhat agree 4**	**Agree Completely 5**
I do not go fast enough to need head protection in a crash					

I feel that helmets are unnecessary for very short rides.					
Being someone who has been riding for years, I can easily avoid an accident when riding					
Motorbike helmets are less important for those who ride their bikes infrequently.					
Motorcycle helmets are more important for those who ride their bikes long distances					
Since I'm not racing or doing any motorbike stunts, I don't really need a helmet.					

Q4b. Perceived Danger of motorcycling

	**Disagree Completely**<br>**1**	**Somewhat disagree**<br>**2**	**Neither agree, nor disagree**<br>**3**	**Somewhat agree**<br>**4**	**Agree Completely**<br>**5**
When I'm riding motorbike, I am at risk of being injured by other bikers					
When I'm riding, I am at risk of being injured by motor vehicles.					
If I had an accident while riding to school or for work and hit my head, I would be likely to suffer brain damage					
Riding motorbikes is dangerous on slippery/wet roads					
There is a good chance that I could get hurt while riding my motorbike					

Q4c Perceived Severity of Harm

		**Disagree Completely**<br>**1**	**Somewhat disagree**<br>**2**	**Neither agree, nor disagree**<br>**3**	**Somewhat agree**<br>**4**	**Agree Completely**<br>**5**
1.	If I injured my head while riding my bike, it could seriously affect my social life with my friends.					
2.	If I injured my head while riding my bike, it could seriously affect my relationships with my family members.					

3.	If I injured my head while riding my bike, it could seriously affect my ability to function at school.					

Q4d. Perceived Benefits

Emotional Benefits

	**Disagree Completely**<br><br>**1**	**Somewhat disagree**<br><br>**2**	**Neither agree, nor disagree**<br>**3**	**Somewhat agree**<br><br>**4**	**Agree Completely**<br><br>**5**
I feel unsafe riding without a helmet					
I feel guilty riding without a helmet.					
Wearing a helmet would make me feel less anxious when I ride a bike.					
I think it is my obligation to keep myself safe for the people who care about me by wearing a helmet when I ride					
Wearing a helmet while riding motorbike makes me feel safer.					
When I wear helmets I feel more aware of the potential dangers of riding					
When I wear helmets I feel more aware of the potential dangers of riding motorbike					

Q4e. Safety Benefits

	**Disagree Completely**<br><br>**1**	**Somewhat disagree**<br><br>**2**	**Neither agree, nor disagree**<br>**3**	**Somewhat agree**<br><br>**4**	**Agree Completely**<br><br>**5**
In general, I think people who choose to wear helmets are being safe and responsible.					
Helmets are effective at reducing my risk of injury during a motorbike-related accident					
The event of an accident, a helmet would protect my head					
In the event of an accident, wearing a helmet could save me					

money by avoiding expensive medical treatment					

Q4f. Perceived Barriers

	**Disagree Completely** **1**	**Somewhat disagree** **2**	**Neither agree, nor disagree** **3**	**Somewhat agree** **4**	**Agree Completely** **5**
The cost of helmets is generally more than they are worth					
The cost of buying a helmet would affect whether I wore one or not.					
The best helmets (that look the coolest and are most comfortable) are too expensive for me to buy					
I would not want to spend money to buy a helmet.					

Q4g.Media

	**Disagree Completely** **1**	**Somewhat disagree** **2**	**Neither agree, nor disagree** **3**	**Somewhat agree** **4**	**Agree Completely** **5**
I recall seeing TV commercials, billboard ads or posters about the importance of wearing a helmet while bike riding during the past year.					
During the past year, I have received advice from my doctor about wearing a helmet while riding motorbike.					
During the past year, I recall seeing magazine ads or newspaper flyers from sporting goods stores or bike shops advertising helmet sales/discounts					
During the past year, I recall some form of helmet use promotion event on campus or in the community					

BEHAVIOR INTENTIONS

During the past 4 weeks , have you tried to use helmet while riding on motorbike?

	Definitely will	Very likely	Quite likely	50/50	Quite unlikel y	Very unlikel y	Definit ely will not
How likely or unlikely is it that you'll be willing to learn about road safety							
How likely or unlikely is it that you'll be wear helmet right away/ immediately (Encourage best guess)							
I am planning to start using helmet within the next months							
How likely or unlikely is it that you'll not use your mobile phone while riding on bike ?							
How likely or unlikely is it that you'll be willing talk to others about road safety and benefits of helmet use?							

THEORY OF PLANNED BEHAVIOR (TPB)

Now I am going to ask your intention in relation to helmet use. How strongly do you disagree or agree with the following statements

Q5a.

		**Disagree Completely**<br><br>**1**	**Somewhat disagree**<br><br>**2**	**Neither agree, nor disagree**<br>**3**	**Somewhat agree**<br><br>**4**	**Agree Completely**<br><br>**5**
Positive attitude	During the past 4 weeks , I have tried to use helmet while riding on motorbike?					
	I feel unsafe when riding without wearing helmet use.					
	Wearing safety helmet while riding					

	motorcycles make me feel safer while driving					
	Safety helmet is an effective equipment for reducing the severity of injuries when accidents occur					
Personal Vanity and Discomfort Barrier-Negative attitude	I feel like a foolish when I wear helmet alone and none wear helmets.					
	I feel that helmet stripes will make me uncomfortable and breathless					
	I would feel embarrassed wearing a helmet.					
	Wearing a helmet is too hot.					
	Wearing a bike helmet strap pinches/would pinch my neck or sometimes irritates my skin					
	A bike helmet strap is uncomfortable, and it feels like I'm being choked					
Subjective norm						
	I feel ashamed if I don't wear safety helmet because my parents pay attention to helmet wearing since I was young					
	My parents taught me to wear helmet when I was young.					
	I am often given praises or acceptance of wearing helmet					

	from family members while riding a motorcycle					
Behavior control and internality	I think that helmet wearing is my own business. It depends on myself either I want to wear or not					
	I can reduce the risk of fatality when the accidents occur by myself when wearing a helmet					
Externaliti es	The risk of accidents depends on myself					
	If I drive bike carefully, the accidents won't occur					
	I think that most motorcycle accidents are caused by road conditions and surroundings					

UNPROMPTED RECALL

Q6a (single choice) During the last **4 weeks**, have you come across any announcements or messages about the road safety or helmet use?

Yes	1	Continue to Q6c-2
No	2	SKIP TO Q6c-3

Q6b (For those who recalled any messages) Can you describe the announcements or messages you saw or heard? (Continue asking): What other messages about the road safety or helmet use did you see or hear? (Write verbatim and continue until respondent can recall no further messages)

Record verbatim the messages or announcements the respondent saw or heard and the channel below:

What else? ____

What else? ____

What else?____

What else? ____

What else? ____

What else? ____

Where respondent recalls a <u>SPECIFIC VIDEO/Channel/Movie/CAMPAIGN MESSAGE</u> - Circle those messages recalled – (Multiple responses allowed): Campaign Messages as follows:

		Yes	No
P1.	Road traffic accident footage	1	2
P2.	Head Injury victim	1	2
P3.	Disability victim (RTA)	1	2
P4.	Deaths due to RTA/ motorbike crashes	1	2
P5.	Traffic violation crime reporting	1	2
P6.	Traffic law enforcement messages	1	2

If one or more campaign messages recalled skip Q3c and go directly to 3D Message Source.

PROMPTED RECALL [GO TO PROMPT CARDS]

Q6c (**Ask all**) During the last 4 weeks, have you come across any of the following announcements or messages about the road safety or helmet use?
(SHOWCARD with single prompts of. – Go through one set of page prompts at a time and ask) Do you remember seeing this message in the last 4 weeks? It may have appeared on media or roads in a number of different forms. (**Then read the header of the prompt cards one by one and record responses**.)

YES: Circle those messages recalled from Prompt Cards: **(Multiple responses allowed)**

		**Yes**	**No**
**P1.**	Road traffic accident footage	1	2
**P2.**	Head Injury victim	1	2
**P3.**	Disability victim (RTA)	1	2
**P4.**	Deaths due to RTA/ motorbike crashes	1	2
**P5.**	Traffic violation crime reporting	1	2
**P6.**	Traffic law enforcement messages	1	2
No campaign messages recalled		2	SKIP TO Q4a.

MESSAGE SOURCE (FOR BOTH UNPROMPTED AND PROMPTED RECALL GROUPS)
Q6d Of the messages or announcements on road safety or helmet use you recall seeing or hearing, do you recall seeing or hearing them from the following sources
[Interviewer note: prompt for the following sources (Multiple choices allowed; randomize sources)

TV	01	Ask 6c
Radio	02	
Billboards	03	

Posters	04	
Newspapers	05	
Magazines	06	
Social Media	07	
Community Drama	08	
Health Workers	09	
Local Leaders	10	
Family Members or Friends	11	
Others (pls specify____)	98	
Others (pls specify____)	98	
Don't Know/Can't Say	95	

MESSAGE TAKEOUT(FOR BOTH UNPROMPTED AND PROMPTED RECALL GROUPS)
Q6e What specific messages did you get from the announcements or messages? What else? What else? (up to 5 messages)

What else? ________________________________

What else? ________________________________

What else? ________________________________

MESSAGE IMPACT
Q6f. After seeing the messages did you?

		No	Yes
**3f_1**	Say anything about them or discuss them with others?	1	2
**3f_2**	Try to persuade others to use helmet while riding motorbike ?	1	2

INTERVENTION EVALUATION

(at the end of session)

Q6g
How strongly do you disagree or agree with the following statements about the messages or announcements you have seen about the road safety & helmet use ?**(Read Out)**

	**Disagree Completely 1**	**Somewhat disagree 2**	**Neither agree, nor disagree 3**	**Somewhat agree 4**	**Agree Completely 5**
The messages were EASY TO UNDERSTAND	1	2	3	4	5
The messages were made me STOP and THINK	1	2	3	4	5

The messages were RELEVANT to me and my life	1	2	3	4	5
The messages PROVIDED NEW INFORMATION to me	1	2	3	4	5
The messages MADE ME FEEL UNCOMFORTABLE	1	2	3	4	5
The messages made me feel CONCERNED ABOUT THE BENEFIT OF USING HELMET	1	2	3	4	5
The messages made me feel CONCERNED ABOUT THE EFFECTS OF MY USING HELMET	1	2	3	4	5
I found some of the IMAGES in the messages DISGUSTING	1	2	3	4	5
The messages made me believe that l am at HIGH RISK of getting injured from motorbike riding	1	2	3	4	5

Thank you for your time and participation in this survey
