# Supplementary material for: Assessment of helmet usage among secondary school students in urban settings: A descriptive analytical study from Karachi, Pakistan
Source: PLoS One. 2026 Jan 9;21(1):e0340608. doi: 10.1371/journal.pone.0340608 (PMC12788624; doi:10.1371/journal.pone.0340608)
Supplement: S2 File — (DOCX) [file pone.0340608.s004.docx]

**PARENTAL/GUARDIAN CONSENT FORM**

**Assessment of Helmet Usage Among Secondary School Students in Urban Settings of Karachi, Pakistan**

**PRINCIPAL INVESTIGATOR**

Mazhar Iqbal, MBBS, MPH

PhD Scholar, School of Public Health

Dow University of Health Sciences, Karachi

Email: mazhar_iqbal17@yahoo.com

**INSTITUTIONAL REVIEW BOARD APPROVAL:**

IRB-2510/DUHS/Approval/2022/856

**INVITATION TO PARTICIPATE**

Your child is invited to participate in a research study on motorcycle helmet use and road safety knowledge among secondary school students in Karachi. Before you decide whether to allow your child to participate, you must understand why the research is being conducted and what it will involve. Please take time to read the following information carefully and discuss it with others if you wish.

**PURPOSE OF THE STUDY**

Road traffic accidents are a leading cause of death and injury among adolescents in Pakistan, with motorcycle riders being particularly vulnerable. Many students in grades 8-10 use motorcycles either as riders or passengers for transportation to school. This study aims to:

1. Assess students' knowledge about road safety rules and helmet usage

2. Understand attitudes and behaviors regarding motorcycle safety

3. Identify barriers to helmet usage among adolescent students

4. Provide evidence to develop effective school-based road safety interventions

Your child has been selected because they are a male student in grades 8-10 (aged 15-19 years) attending a public secondary school in Karachi and use a motorcycle for transportation.

**WHY HAS MY CHILD BEEN INVITED?**

Approximately 500 students from 10 public secondary schools across Karachi will participate in this study. Your child has been randomly selected from students who meet the following criteria:

- Male student aged 15-19 years

- Currently enrolled in grades 8-10

- Uses a motorcycle for transportation (as rider, pillion rider, or passenger)

**DOES MY CHILD HAVE TO TAKE PART?**

No Participation in this study is completely voluntary. Your child does not have to participate if they do not wish to do so. You are free to withdraw your consent at any time without giving a reason. If you decide not to allow your child to participate or if you withdraw consent later, this will NOT affect:

- Your child's education or standing at school

- Your child's relationship with teachers or school administration

- Your family's access to any services

WHAT WILL HAPPEN IF MY CHILD TAKES PART?

If you agree to allow your child to participate:

1. Interview Session (One-time, approximately 30-40 minutes)

- A trained researcher will meet with your child at school during school hours

- The interview will be conducted in a private, comfortable setting

- Your child will be asked questions about:

- Their demographics (age, family background, socioeconomic status)

- Transportation methods used for school

- Knowledge about road safety rules and traffic signs

- Attitudes and beliefs about helmet usage

- Current practices regarding helmet wearing

- Influences from family and friends on safety behaviours

2. Language

- The questionnaire will be administered in Urdu to ensure your child understands all questions

- Researchers will be available to clarify any questions

WHAT ARE THE POSSIBLE BENEFITS?

Direct Benefits to Your Child:

- Increased awareness about road safety and importance of helmet usage

- Opportunity to contribute to improving road safety for students in Pakistan

Benefits to Society:

- Better understanding of barriers to helmet usage among adolescents

- Evidence to develop effective school-based road safety programs

- Potential to reduce motorcycle-related injuries and deaths among young people

WHAT ARE THE POSSIBLE RISKS OR DISADVANTAGES?

The risks associated with this study are minimal:

- Time commitment: Approximately 30-40 minutes during school hours
- Trained interviewers will maintain a supportive, non-judgmental approach
- Your child can take breaks during the interview if needed
- The interview will be stopped immediately if your child becomes distressed

WILL MY CHILD'S PARTICIPATION BE KEPT CONFIDENTIAL?

Yes, absolutely. We are committed to protecting your child's privacy:

Confidentiality Measures:

1. No Identifying Information:

- Your child will be assigned a unique identification number

- Names will NOT be recorded on questionnaires

- School identifiers will be kept separate from study data

2. Secure Data Storage:

- All paper questionnaires will be kept in locked filing cabinets

- Electronic data will be stored on password-protected computers

- Only the research team will have access to the data

- Data will be stored for the required period as per institutional policy

3. Anonymized Reporting:

- Results will be presented as group averages and percentages

- No individual student will be identifiable in any reports or publications

- Schools will only receive aggregate data, not individual information

4. Limits to Confidentiality:

- If your child discloses information suggesting immediate danger to themselves or others, we are ethically obligated to report this to appropriate authorities

WHAT WILL HAPPEN TO THE RESULTS OF THE STUDY?

Publication and Dissemination:

- Results will be published in scientific journals and presented at conferences

- A summary of findings will be shared with participating schools and education authorities

- Results may inform road safety policy and school-based interventions

Access to Results:

- You can request a summary of the study findings by contacting the research team

- No individual results will be provided as data is analysed collectively

Funding: This study received no external funding and is conducted as part of doctoral research at Dow University of Health Sciences.

WHO HAS REVIEWED THIS STUDY?

This study has been reviewed and approved by:

Institutional Review Board

Dow University of Health Sciences

Approval Number: IRB-2510/DUHS/Approval/2022/856

The IRB has confirmed that:

- The study design is ethically sound

- Risks to participants are minimized

- Confidentiality protections are adequate

- The informed consent process is appropriate

WHAT IF THERE IS A PROBLEM?

Complaints or Concerns:

If you have any complaints or concerns about any aspect of this study, please contact:

Primary Contact:

Mazhar Iqbal, MBBS, MPH

Phone: [0XXXXXXXX]

Email: mazhar_iqbal17@yahoo.com

**CONSENT STATEMENT**

I have read and understood the information provided above. I have been allowed to ask questions and my questions have been answered to my satisfaction.

I understand that:

- My child's participation is voluntary

- My child can withdraw at any time without giving a reason

- My child's information will be kept confidential

- My child's participation will not affect their education or school standing

- The results may be published but my child will not be identified

I voluntarily agree to allow my child to participate in this research study.

**PARENT/GUARDIAN INFORMATION:**

Name of Parent/Guardian: _________________________________

Signature: _________________________________ Date: _______________

Relationship to Child: _________________________________

Contact Number: _________________________________

**CHILD/STUDENT INFORMATION:**

Name of Student: _________________________________ (PRINT)

Age: _________ Grade: _________

School Name: _________________________________

STUDENT CONSENT (If student is 18 years or older):

I agree to participate in this study.

Student Signature: _________________________________ Date _______________

RESEARCHER STATEMENT:

I have explained the nature and purpose of this study to the parent/guardian named above. I have answered all questions to the best of my ability.

Name of Researcher: _________________________________

Signature: _________________________________Date: _______________

FOR OFFICE USE ONLY:

Participant ID Number: _________________________________

Date of Consent: _________________________________
